# Stratified vector control and proactive cross border collaboration for sustaining malaria elimination in Yunnan, China

**DOI:** 10.1136/bmj-2024-082300

**Published:** 2025-04-22

**Authors:** Dashan Zheng, Peng Tian, Guiyun Yan, Zurui Lin, Hongning Zhou, Xiaobo Liu, Jianheng Chen, Qiyong Liu, Hualiang Lin

**Affiliations:** 1 Joint International Research Laboratory of Environment and Health, Ministry of Education, Department of Epidemiology, School of Public Health, Sun Yat-sen University, Guangzhou, Guangdong, China; 2 Yunnan International Joint Laboratory of Tropical Infectious Diseases and Yunan Key Laboratory of Insect-borne Infectious Diseases Control of Yunnan Institute of Parasitic Diseases, Puer, Yunnan, China; 3 School of Population and Public Health, University of California, Irvine, CA, USA.; 4 National Key Laboratory of Intelligent Tracking and Forecasting for Infectious Diseases, National Institute for Communicable Disease Control and Prevention, Chinese Center for Disease Control and Prevention; WHO Collaborating Centre for Vector Surveillance and Management, Beijing, China; 5 School of Public Health, Nanjing Medical University, Nanjing, Jiangsu, China

## Abstract

**Hualiang Lin and colleagues** emphasise that achieving and sustaining malaria elimination in border areas requires stratified risk management, targeted vector control, timely epidemiological surveillance and response, environmental improvement, and cross border collaboration for joint prevention and control

Yunnan Province was China’s last region to eliminate indigenous malaria transmission owing to its extensive, porous borders with three malaria endemic countries—Myanmar, Laos, and Vietnam.^1^ The province faced immense pressure to control imported cases of malaria, particularly during the early years of China’s reform and opening-up in the 1980s.^2^ Moreover, the complex malaria epidemiology, diverse ecological features, multiple vector species, and underdeveloped economic environment presented significant challenges.

Globally, many countries face similar challenges in controlling cross border malaria transmission. Frequent population movement in border areas, inequalities in healthcare resources between neighbouring countries, and the complexity of cross border collaboration make effectively controlling and eliminating malaria difficult for one single country. For instance, countries in the Greater Mekong Subregion have long struggled with imported malaria cases, as labour migration and trade activities exacerbate cross border malaria transmission.^3^ ^4^ Similarly, border regions in Africa, such as those between Kenya and Tanzania, face difficulties in implementing unified and effective interventions owing to differences in malaria control strategies and resource allocation across countries.^5^ Tackling cross border malaria transmission is a pressing global priority requiring experiences from countries and regions with successful elimination.

In the 1980s the Chinese government introduced the ambitious goal of elimination of malaria.^6^ To achieve this goal, Yunnan province implemented border specific control strategies that incorporated many of the technical measures used throughout China’s malaria elimination efforts from 1980 to the present (fig 1). These tailored approaches in Yunnan are particularly noteworthy given Yunnan’s unique challenges as a high burden border region characterised by multiple vector species and imported cases, both from travel to malaria endemic areas and cross border mosquito transmission. The successful malaria elimination in Yunnan provides valuable experiences for global malaria control and elimination efforts, especially in bordering areas facing similar cross border challenges.^7^ By tackling the complexities of border dynamics and tailoring interventions to local contexts, Yunnan offers insights for how malaria can be eliminated and its reintroduction prevented in areas where the risks are greatest.

**Fig 1:**
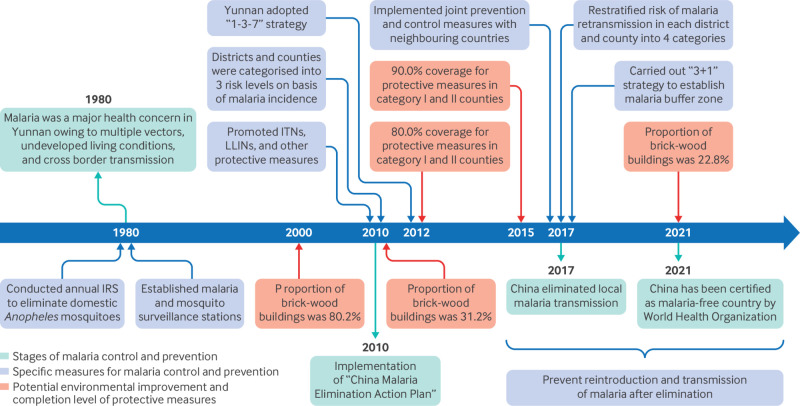
Timeline of malaria control in Yunnan, China, 1980-2023. IRS= indoor residual spraying; ITN=insecticide treated bed nets; LLIN=long lasting insecticidal nets

## Malaria in Yunnan: multiple vectors, living conditions, and cross border transmission

Compared with other regions in China, eliminating malaria in Yunnan was more challenging.^3^ Yunnan is home to multiple vector species including *Anopheles sinensis*, *An minimus*, *An dirus*, and *An lesteri*,^8^ which have different feeding and resting behaviours and require integrated and sustainable measures for control. In addition, owing to economic constraints and the traditional practice of some ethnic groups, many housing structures were constructed using wood and bamboo,^9^ which inadvertently created environments conducive to the breeding and growth of mosquitoes. Importantly, Yunnan also shares a 4060 km land border with three malaria endemic countries—Myanmar, Laos, and Vietnam—within the Greater Mekong Subregion,^10^ which is a major risk factor for transmission.

**Fig 2:**
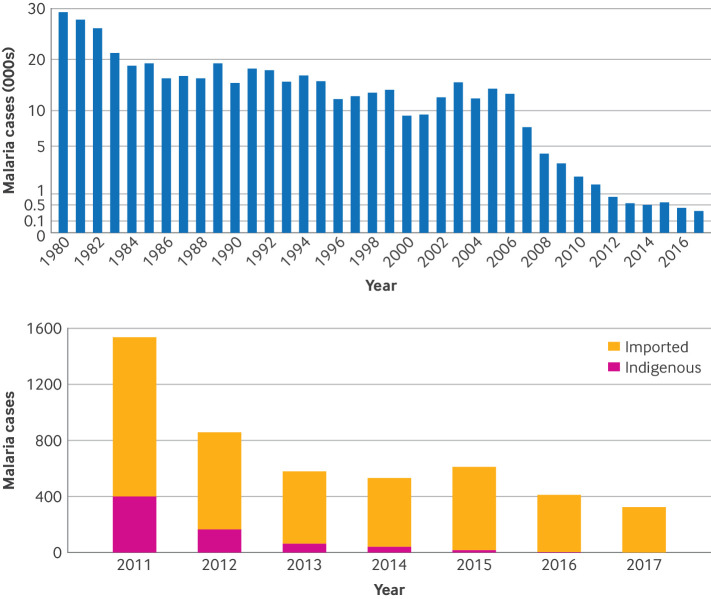
Time series of malaria cases in Yunnan, 1980-2017. Top plot shows trend of malaria cases, with bottom plot highlighting imported and indigenous cases in Yunnan from 2011 to 2017

From 1980 to 2023, a total of 472 112 cases of malaria were reported in Yunnan,^11^ with a steady declining trend (fig 2).^12^ However, most malaria outbreaks in Yunnan were attributed to imported cases or cross border movement of infected *Anopheles* mosquitoes.^13^ Between 2011 and 2023, 688 indigenous cases (locally transmitted cases or cases without evidence of importation) were reported, whereas 5394 (88.7%) cases were imported,^11^ highlighting cross border transmission as the primary risk factor for malaria’s reappearance in Yunnan.

## Key strategies for eliminating malaria in Yunnan (1980-2016)

### Malaria control strategies over three decades after reform

Since 1980, China’s reform and opening-up policies have facilitated economic growth, cross border trade, and labour migration, which contributed to a sharp increase in cross border population movement in Yunnan, increasing the importation and spreading of malaria cases. Recognising this challenge, Yunnan’s provincial government took direct leadership and implemented a series of malaria control policies to tackle both local and cross border risks. For instance, a malaria control training programme was launched, which trained approximately 35 800 primary health practitioners annually between 1980 and 1998 to enhance local response capacity.^2^ Yunnan’s provincial health authorities also implemented a mass preventive medication campaign under the support of the Chinese government to curb malaria in cities with severe outbreaks. Yunnan also established a mosquito surveillance system to collect data on *Anopheles* mosquitoes and did research to improve vector control and enhance early detection of malaria cases. These measures reflect a comprehensive approach to managing the unique challenges posed by Yunnan’s increasing cross border dynamics.

### Stratified risk management and “1-3-7” strategy

**Fig 3:**
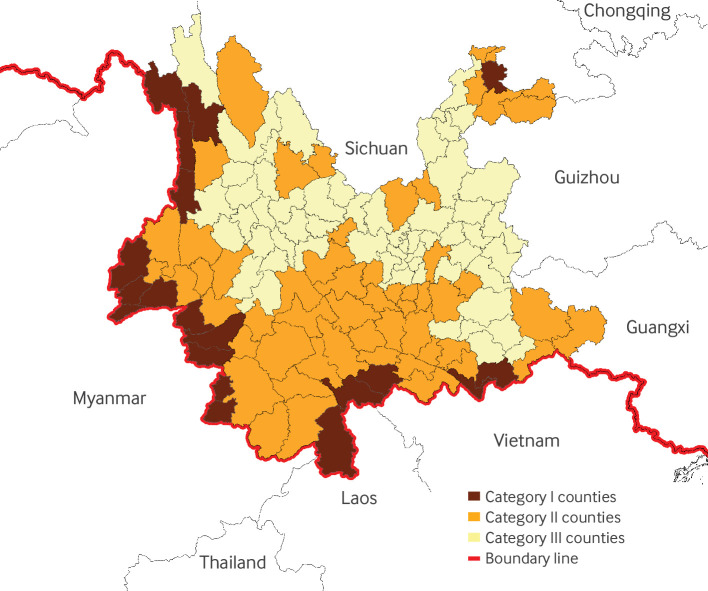
Spatial distribution of three malaria risk categories

Building on the previous three decades of malaria control strategies, the Chinese government further launched the China Malaria Elimination Action Plan (CMEAP) in 2010 to enhance the elimination efforts.^14^ According to this plan, districts and counties in Yunnan were classified into three risk levels on the basis of malaria incidence and were required to conduct stratified risk management (fig 3).^15^ Counties were classified into category I if indigenous malaria cases occurred every year in 2006-08 and the annual incidence rate was not lower than 0.01%. These counties were required to achieve an epidemic management rate of 50% by 2012 and 100% by 2015. Local Centers for Disease Control and Prevention (CDCs) were responsible for conducting case searches, testing blood samples from individuals with a two week fever history, and implementing vector control measures at outbreak sites. Category II counties were those that had reported indigenous cases during the three years, with an incidence rate lower than 0.01% for at least one year in 2006-08; targets were set to achieve a management rate of 70% by 2012 and 100% by 2015. Category III counties were those without any indigenous cases for the three years and were required to consistently maintain a management rate of 100%.

As part of implementing CMEAP, Yunnan’s provincial government implemented a “1-3-7” strategy in 2012. This approach, designed to prevent the spread of imported cases across China,^16^ involved a systematic timeline: reporting and treating cases within one day of diagnosis, completing case investigations within three days after reporting, and conducting focal epidemic management within seven days to curb secondary malaria transmission.^16^

### Targeted vector control measures and living environment improvement

Although imported cases were the primary concern, local strategies also targeted Yunnan’s diverse malaria vectors, including sylvan, domestic, and peridomestic species whose behavioural variability required multifaceted interventions.^17^

*An minimus* is the primary domestic mosquito species in Yunnan, with approximately 20 times the transmission capacity of *An sinensis*.^17^ Since 1980 Yunnan has conducted annual indoor residual spraying once or twice annually. Insecticide treated bed nets and long lasting insecticidal nets treated with pyrethroid insecticides were also widely distributed following CMEAP.^18^ ^19^ By 2012 coverage of insecticide treated bed nets and long lasting insecticidal nets had reached 80% in category I and II counties, and this increased to 90% by 2015 (fig 1).^15^

*An sinensis*, the most common peridomestic malaria vector, feeds around human dwellings and lays eggs in shallow waters such as rice fields. Thus, Yunnan adopted the rice-fish farming strategy and had reduced its larval density by up to 80%.^20^ Sylvan mosquitoes were controlled with insecticide applications to tropical vegetation around villages and providing outdoor workers with repellents, prophylactic drugs, and long lasting insecticidal nets free of charge.^21^ ^22^

Improving housing structures also played a critical role in reducing human-mosquito contacts. Traditional wooden and bamboo dwellings facilitated mosquito entry owing to the gaps in the eaves and walls and thus increased the risk of malaria transmission.^23^ Alongside the economic development, Yunnan’s provincial government facilitated the adoption of concrete structures, reducing the proportion of buildings made of these materials in Yunnan from 80.2% in 2000 to 22.8% in 2020.^24^ Furthermore, the built-up area increased from 1200.4 km^2^ in 2008 to 2012.9 km^2^ in 2022 (fig 1), which effectively reduced mosquito breeding grounds.^25^ ^26^

### Overcoming challenges of insecticide resistance in malaria elimination

Since 1980, Yunnan has achieved notable success in malaria control. However, long term reliance on single insecticides led to resistance among mosquitoes, presenting ongoing challenges.^27^ ^28^ To tackle this problem, Yunnan CDC phased out dichlorodiphenyltrichloroethane (DDT), replacing with organophosphates, then deltamethrin, and eventually the mixture of propoxur (6%) and β-cypermethrin (4%) by 2020. The province also monitors insecticide resistance and prepares alternative insecticides to respond to potential resistance to β-cypermethrin.^24^ ^29^

Insecticide spraying is an affordable and efficient method often favoured by low and middle income countries with severe malaria burdens, especially in Africa and South East Asia.^30^ ^31^ The experience and lessons in Yunnan highlight the importance of continuous resistance monitoring, timely insecticide replacement, and use of insecticide mixtures to maintain the efficacy of residual spraying.

## Key strategies for preventing cross border retransmission of malaria in Yunnan (2017-23)

Since 2017, malaria cases in Yunnan have been attributed to cross border imported cases (fig 2). Thus, with the guidance of the Chinese government, Yunnan CDC transitioned its focus from tackling both indigenous and imported malaria cases to prioritising the detection and prevention of imported cases, which is one key strategy to sustain malaria elimination.^32^

### Re-stratification of malaria retransmission risk

**Fig 4:**
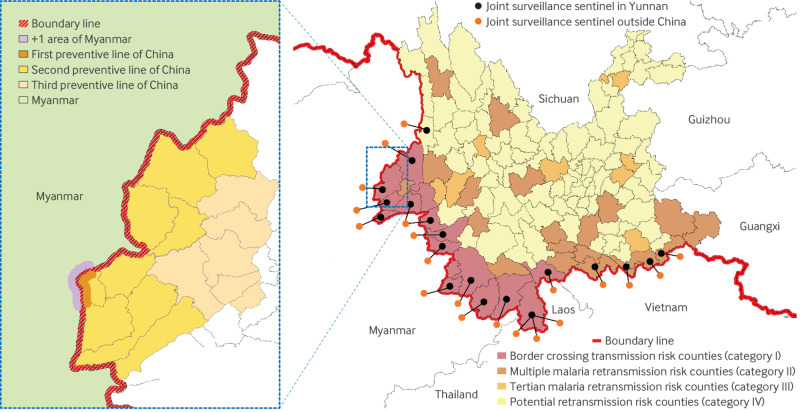
Re-stratification of malaria retransmission risk in Yunnan and joint “3+1” cross border control strategy. Left side shows distribution of counties within Yunnan’s three preventive lines and border areas under “3+1” strategy. Right side shows re-stratification of malaria retransmission risk and distribution of sentinel sites for cross border joint surveillance, prevention, and control within China, as well as in Myanmar, Laos, and Vietnam

Following the elimination of indigenous cases, Yunnan re-stratified the malaria transmission risk into four categories (I-IV), from high to low, according to the types of imported cases and mosquito vectors (fig 4).^33^ Category I counties are those with local vectors in the previous three years and adjacent to malaria endemic regions. In these counties, all township health centres are mandated to test (via rapid diagnostic tests or microscopy) all febrile patients and screen individuals who have been active within 2.5 km of the border. Category II refers to counties with multiple vector species such as *An minimus* and *An lesteri*, capable of transmitting *Plasmodium vivax* and *P falciparum*. In category II counties, all township health centres are required to test febrile patients with a history of travel to malaria endemic areas within the previous two years or previous malaria infection. Category III is defined as counties that have only *An sinensis* and imported *P vivax*, and category IV includes counties without imported cases. In category III-IV counties, only selected township health centres are required to test febrile patients meeting the same criteria as those in category II.

### Proactive joint sentinel monitoring along cross border areas

Besides the continuous surveillance for mosquito species and density, and insecticide resistance in Yunnan, proactive joint prevention and control measures were also initiated by establishing cooperative sentinel monitoring sites in neighbouring countries along cross border areas. As of 2023, 20 sentinel sites have been established in Myanmar (13 sites), Laos (four sites), and Vietnam (four sites), corresponding with 19 sentinel sites in Yunnan (fig 4, right side). Each sentinel site in these three countries was paired with a domestic site in Yunnan. All the sites were managed by the corresponding counties within Yunnan.^34^ These sites were responsible for simultaneous vector monitoring, technology sharing (such as detection technologies, effective drugs, insecticides, and equipment), and organising regular information exchanges on local epidemic updates and recent prevention experiences.^34^

### Joint “3+1” strategy along China-Myanmar border areas

Since 2017 China has implemented joint prevention and control measures with Vietnam and Laos, focusing mainly on establishing cooperative sentinel sites. Collaboration with Myanmar has been more comprehensive owing to its higher malaria burden, longer shared border, and less developed health infrastructure, with the “3+1” strategy being implemented (fig 4).^35^ The “3” refers to three preventive lines, and “1” represents villages, communities, and settlements within 2.5 km of the international border outside the territory of China. In “+1” areas, joint task forces implemented comprehensive malaria control measures. The objective was to strengthen Myanmar’s malaria control programmes through financial and technical support, capacity building and training, vector monitoring and control, health education, and treatment guidance.

In regions with the “3+1” strategy, real time monitoring of malaria cases and timely intervention measures were conducted. These included blood examinations, provision of medication, indoor residual spraying coverage, and epidemic investigation and interventions. Sixty six malaria control consultation and service stations were established in 19 counties bordering Myanmar to promptly treat malaria cases, administer health education knowledge, and provide long lasting insecticidal nets for the mobile Chinese population.^36^

### Lessons on sustaining malaria elimination in the cross border context

On the basis of malaria data (1980-2023) from the Yunnan Institute of Parasitic Diseases, we did an interrupted time series analysis and found that more than 8400 cases of malaria were successfully prevented following the launch of CMEAP in 2010. Yunnan’s approach to malaria elimination possesses several key elements: the leadership of the national and local government emphasising the management of imported cases, and steady cross border collaboration.

However, challenges such as political instability and conflicts in neighbouring countries could threaten the long term sustainability of malaria elimination, particularly by undermining cross border collaboration.^37^ Since 2021 the armed conflict in Myanmar has complicated efforts to sustain malaria elimination in Yunnan. Conflict induced economic turmoil in Myanmar disrupted access to antimalarial drugs,^38^ creating an insufficient workforce for local malaria control measures. Additionally, the movement of refugees towards the Chinese border heightened the risk of potential retransmission in Yunnan.^39^ The conflict has also strained Myanmar’s resources, leading to challenges to sustaining joint prevention and control measures. To alleviate the pressure on the joint measures, the Yunnan Institute of Parasitic Diseases dispatched more public health professionals to support joint collaboration in Myanmar.

Yunnan’s comprehensive joint prevention measures are currently focused on Myanmar. However, strengthening collaboration with other Lancang-Mekong countries is equally critical, as these countries are also major sources of imported malaria cases in China. The Chinese government will continue implementing the “1-3-7” strategy in Yunnan and provide consistent funding to strengthen joint efforts with Lancang-Mekong countries. This includes fostering stronger cross border collaborations with countries in the Lancang-Mekong River Basin. In 2019 a joint prevention and control platform was established to facilitate the acquisition and sharing of vector and malaria surveillance data, meteorological information, and socio-cultural data.^40^ This collaboration also established multiple fever clinic surveillance stations along the China-Laos border and developed a surveillance network covering five northern provinces of Laos.^40^

The successful elimination of malaria in Yunnan, China, despite diverse malaria vector species and cross border transmission, provides valuable experience for achieving and sustaining malaria elimination in other regions, especially the importance of stratified risk management, targeted vector control, timely insecticide replacement, cross border collaboration with stable political conditions, and strong partnerships.

**Key messages**

Eliminating malaria in bordering areas was distinctive and challenging owing to multiple vector species, underdeveloped living environment, and cross border transmissions
The successful malaria elimination in Yunnan was mainly attributed to stratified risk management, integrated environment management, targeted vector control, and timely epidemiological survey and response.
Key strategies for sustaining malaria elimination included proactive cross border collaboration, precise surveillance, and timely response
Besides the strategies and measures to eliminate malaria in China, Yunnan offers some unique insights for effectively managing malaria in bordering areas

## Notes

Contributors and sources: DZ and PT contributed equally as first authors. QL and HL conceived, designed, and supervised this analysis. DZ and PT collected and analysed data and wrote the paper. ZL and HZ were responsible for data collection. GY and XL drafted and revised the manuscript. JC was responsible for the figures and data analysis. QL (liuqiyong@icdc.cn) and HL (linhualiang@mail.sysu.edu.cn) contributed equally to this work and should be considered co-corresponding authors. All authors read and approved the manuscript.

Provenance and peer review: Commissioned; externally peer reviewed.

This article is part of a collection developed in partnership with the Chinese Center for Disease Control and Prevention, Beijing. Open access fees were funded by the Gates Foundation, China. *The BMJ* commissioned, peer reviewed, edited, and made the decision to publish. Rachael Hinton and Jocalyn Clark were the lead editors for *The BMJ*.
